# Modulation of Intestinal Barrier Function and Wnt/β-Catenin Pathway by Sanzi Formula in Colorectal Adenoma Development

**DOI:** 10.5152/tjg.2025.24712

**Published:** 2025-04-07

**Authors:** Wanqiu Peng, Zhongyi Li, Shuting Zou, Hui Li, Yaozhou Tian, Yongzhi Hua, Lanfu Wei, Zhenhai Zhang, Yi Gu, Tingting Xu

**Affiliations:** 1 Affiliated Hospital of Integrated Traditional Chinese and Western Medicine, Nanjing University of Chinese Medicine, Jiangsu, China

**Keywords:** Wnt/β-catenin signaling pathway, colorectal adenoma, inflammatory factors, intestinal barrier function, Sanzi formula

## Abstract

**Background/Aims:**

Colorectal adenoma is a high-risk precursor lesion of colorectal cancer. Sanzi formula (SZF), a traditional Chinese medicine compound for the treatment of this disease, inhibits the growth of colorectal adenomas. This study aimed to investigate the mechanism by which SZF blocks the growth of colorectal adenomas in Apc^min/+^ mice.

**Materials and Methods:**

C57BL/6J mice were used as the control group and were fed a normal diet. To create the colorectal adenoma model, Apc^min/+^ mice were fed high-fat chow. Each administration group was given the appropriate drug by gavage at the same time as the modeling. The body weight, adenoma size, and adenoma number of mice in each group were recorded, and the colorectal tissue was subjected to HE staining and histopathological evaluation. The effect of SZF on inflammatory factors, tight junction (TJ) proteins, pathway proteins, and mRNA content in colorectal adenoma was examined by enzyme-linked immunosorbent assay, western blotting, immunofluorescence analysis, and quantitative real-time polymerase chain reaction.

**Results:**

The results showed that all dose groups of SZF improved the size, number, and histopathological damage of adenomas. All dose groups of SZF resulted in lower inflammatory factors, higher levels of TJ proteins, and lower levels of pathway proteins and mRNA. Particularly, the high-dose group of SZF was more effective.

**Conclusion:**

Sanzi formula interfered with the progression of colorectal adenoma in Apc^min/+^ mice by repairing the intestinal barrier function and blocking the Wnt/β-catenin signaling pathway, which provided a new idea and scientific basis for clinical treatment.

**Main Points**

- This study investigated the mechanism by which Sanzi formula (SZF) prevents the growth of colorectal adenomas in Apc^min/+^ mice.
- The number and diameter of colorectal adenomas were reduced in each of the SZF treatment groups compared with the model group.
- Sanzi formula inhibited the abnormal activation of the Wnt/β-catenin pathway, reduced inflammation, and increased tight junction protein content.

## Introduction

Colorectal adenoma is a benign tumor in the mucosal epithelium of the colorectal area. Adenomas are classified into different types, such as tubular adenomas, villous adenomas, and tubular villous adenomas, based on their histologic morphology.^1^ Most patients with colorectal adenoma have no obvious symptoms. However, adenomas that are large in diameter, irregular in shape, have ulcers or bleeding on the surface, and have a pathologic type of villi or tubular villi may develop into colorectal cancer. Colorectal cancer has the second highest mortality rate in the world, posing a serious challenge to public health. Currently, the main clinical treatments for colorectal adenoma are medication and surgery. The use of drug treatment, such as nonsteroidal anti-inflammatory drugs, can only slow down the growth of colorectal adenomas and can’t completely eradicate them. Surgical treatment, on the other hand, is difficult and involves risks such as perforation and bleeding, and patients have a long postoperative recovery time.^2^^,^^3^ Therefore, it is urgent to explore a safer and more effective treatment for colorectal adenoma.

Traditional Chinese medicine has demonstrated unique advantages and potential in cancer treatment. Among them, the ethnodrug Sanzi formula (SZF) from the experience of Yaozhou Tian has demonstrated significant clinical efficacy in treating colorectal adenoma and early colorectal cancer. Sanzi formula is composed of *Astragali Radix*, *Mume Fructus*, *Semen Punicae Granati*, *Entadae Semen*, *Phyllanthi Fructus*, *Zanthoxyli Pericarpium*, *Uncariae Ramulus Cum Uncis*, *Curcumae Radix*, and *Glycyrrhizae Radix et Rhizoma*. Recent research in pharmacology has demonstrated that SZF possesses anti-inflammatory, anti-tumor, antioxidant, and immune-suppressive properties.^4^ In addition, the team carried out a clinical study on the prevention and treatment of adenomatous colorectal polyp recurrence by SZF in the hospital. The results showed that SZF could reduce the adenoma recurrence rate from 26.6% to 13.1% and attenuate patients’ symptoms such as abdominal pain, abdominal distension, constipation, diarrhea, and a bitter taste in the mouth. However, the mechanism of inhibiting the development of colorectal adenoma by SZF is still not well understood and requires in-depth study.

Wnt/β-catenin is an incredibly conserved signaling system that is essential to the growth and development of organisms in biological evolution.^5^ The Wnt/β-catenin signaling system has a major impact on tumorigenesis, progression, metastasis, and the emergence of therapeutic resistance. *Helicobacter pylori* infection stimulates gastric cancer development by blocking iron death through activation of the Wnt/β-catenin signaling pathway. The findings suggest that blocking this signaling pathway specifically increases the vulnerability of gastric cancer cells to iron death.^6^ Aberrant activation of this pathway is considered to be an important driver for tumor formation and progression. Therefore, finding strategies to inhibit the Wnt/β-catenin signaling pathway’s aberrant activation is crucial for preventing and treating colorectal adenomas.

It was speculated that the growth of colorectal adenomas in Apc^min/+^ mice may be inhibited by SZF through the regulation of intestinal barrier functional integrity and the Wnt/β-catenin signaling pathway. The mechanism of SZF’s role in these processes was revealed, contributing to the development of new prevention and therapeutic strategies for colorectal adenoma.

## Materials and Methods

### Reagents and Chemicals

High-fat feed (model number: D12492) was obtained from the Research Diets, Inc. (USA). *Astragali Radix*, *Mume Fructus*, *Semen Punicae Granati*, *Entadae Semen*, *Phyllanthi Fructus*, *Zanthoxyli Pericarpium*, *Uncariae Ramulus Cum Uncis*, *Curcumae Radix*, and *Glycyrrhizae Radix et Rhizoma* were offered by the Affiliated Hospital of Integrated Traditional Chinese and Western Medicine, Nanjing University of Chinese Medicine. TNF-α (Catalog Number: H052-1-1), Interleukin-6 (IL-6) (Catalog Number: H007-1-4), and IL-1β (Catalog Number: H002-1-1) enzyme-linked immunosorbent assay (ELISA) kits were purchased from Nanjing Jiancheng Bioengineering Institute Co., Ltd. (Nanjing, China). Zonula occluden-1 (ZO-1) (Catalog Number: 21773-1-AP), occludin (Catalog Number: ab216327), claudin-1 (Catalog Number: 28674-1-AP), β-catenin (Catalog Number: 51067-2-AP), cyclin D1 (Catalog Number: ab134175), and c-myc (Catalog Number: 10828-1-AP) antibodies were supplied by Proteintech Group, Inc. (Wuhan, China). Four percent paraformaldehyde (Catalog Number: G1101-500ML) was provided by Servicebio, Inc. (Wuhan, China).BCA protein concentration determination kit (Catalog Number: AR0146) was provided by Wuhan Boster Biological Technology Co., Ltd. (Wuhan, China).

### Experimental Animals

Apc^min+^ mice are able to spontaneously generate multiple colorectal adenomas, making them an ideal colorectal tumor model.^7^^,^^8^ Ten SPF-grade 7-week-old C57BL/6J male mice (20.0 ± 2.0 g) and 40 SPF-grade 7-week-old Apc^min/+^ male mice (20.0 ± 2.0 g) were obtained from Changzhou Cavens Experimental Animal Co., Ltd. under certificate of conformity No. 202254103. The trial was carried out in the Jiangsu Province Academy of Traditional Chinese Medicine’s Animal Experimental Center, where the animals were kept at 25 ± 2°C and 55 ± 5% relative humidity with drinking and eating freely. The Animal Ethics Committee of Jiangsu Provincial Academy of Chinese Medicine authorized the experimental protocol (approval number: AEWC-20220622-219, date: June 22, 2022). This study does not involve human participants, and therefore, informed consent was not required.

### Preparation of Sanzi Formula Extracts

Sanzi formula is composed of *Astragali Radix* 10 g, *Mume Fructus* 10 g, *Semen Punicae Granati* 10 g, *Entadae Semen* 10 g, *Phyllanthi Fructus* 10 g, *Zanthoxyli Pericarpium* 3 g, *Uncariae Ramulus Cum Uncis* 10 g, *Curcumae Radix* 10 g, and *Glycyrrhizae Radix et Rhizoma* 3 g. All the dried herbs were soaked in distilled water (10 times the dose) for half an hour. The herbs were boiled in distilled water, kept at a light boil for 2 hours, and filtered. This extraction step was repeated 3 times. The filtrates were combined, concentrated to 0.3 g/mL, and stored at 4°C.

### Establishment of a Mouse Model of Colorectal Adenoma, Administration, and Sample Collection

After 1 week of acclimatization feeding, C57BL/6J mice were fed normal food and water as a control group, and Apc^min/+^ mice were fed high-fat chow for 20 weeks with normal water intake to establish a colorectal adenoma mouse model. Apc^min/+^ mice were assigned, according to randomization, into 4 different groups: model group, low-dose SZF (L-SZF, 1.7 g/kg), medium-dose SZF (M-SZF, 3.4 g/kg), and high-dose SZF (H-SZF, 6.8 g/kg). The dosage of SZF was based on the daily dose for patients in clinical practice (20g/70kg), converted according to the dosage administered between humans and animals, and was the intermediate dosage of this experiment.

From Monday through Friday, the SZF dosage groups received their corresponding medications intragastrically, while the model and control groups received saline intragastrically. Meanwhile, the weight fluctuations of the mice in all groups were noted every Monday.

At the end of the trial, euthanasia was performed by rapid cervical dislocation after isoflurane inhalation anesthesia. The mice in each group were dissected to take their proximal anal colon and rectum. Adenoma formation was observed, and the number and size of colorectal adenomas were analyzed. Subsequently, a portion of the colorectal tissue was immersed in 4% paraformaldehyde, and the other portion was frozen at −80°C for subsequent analysis.

### Hematoxylin and Eosin Staining of Colorectal Adenoma Tissue

Colorectal tissue was fixed with 4% paraformaldehyde, embedded in paraffin, and sliced into 5 μm-thick sections. The slices were first deparaffinized using a gradient of xylene and ethanol, then stained with hematoxylin and eosin (H&E), and placed under a light microscope to assess the pathological changes of the colorectal tissue.

### Enzyme-Linked Immunosorbent Assay Kit Detection

The appropriate amount of colorectal tissue was weighed to remove blood and impurities using pre-cooled PBS. The tissue was homogenized on ice. It was then centrifuged at 9600 x g for 15 minutes at 4°C. The supernatant was collected, and the colorectal tissue was assayed for IL-6, IL-1β, and TNF-α according to the instructions of the ELISA kit.

### Western Blot Detection

The colorectal tissue was well sheared and added to RIPA buffer containing 1% PMSF for homogenization, lysed on ice for half an hour, followed by centrifugation at 4°C at 10 000 rpm for 10 minutes, and collection of the supernatant. Total protein concentration was determined using a BCA protein concentration determination kit . Proteins were separated using SDS-PAGE electrophoresis, electrotransferred to PVDF membranes, and incubated overnight at 4°C with primary antibody solutions. After washing the outer layers of the membranes with TBST, the membranes were treated with secondary antibody for 1 hour at room temperature, shielded from light. After washing the membranes, the color was developed with enhanced chemiluminescence reagent, the images were captured with a chemiluminescence imaging system, and the protein gray values were analyzed using Image J-win64 (National Institutes of Health, Bethesda, MD, United States).

### Immunofluorescence Assay

Colorectal tissue sections were processed for antigen retrieval, then sealed with bovine serum albumin for 1 hour and incubated dropwise with ZO-1, Occludin, and Claudin-1 primary antibodies overnight at 4°C. A fluorescent secondary antibody was incubated for 1 hour at room temperature away from light. Then, nuclei were counterstained with DAPI. Finally, the images were acquired using a fluorescence microscope.

### Quantitative Real-Time Polymerase Chain Reaction Analysis

Total colorectal RNA was obtained from colorectal tissue using TRIzol reagent, and cDNA was synthesized by reverse transcription, and then the target gene was amplified using cDNA as a template. The relative expression of β-catenin, cyclin D1, and c-myc mRNA was calculated using the 2^-ΔΔCt^ method with GAPDH as an internal reference. The list of specific primers is shown in Table 1.

### Statistical Analysis

Statistical analysis was conducted using SPSS 26.0 (IBM SPSS Corp.; Armonk, NY, USA), with results presented as mean ± standard deviation (x̄ ± SD). Comparisons between 2 groups were performed using unpaired *t*-tests; comparisons among multiple groups were performed using one-way analysis of variance (ANOVA), and further two-by-two comparisons were performed using LSD tests. *P* < .05 was considered a statistically significant difference. All graphical representations were generated using GraphPad Prism 8.3.0 (GraphPad Software, San Diego, CA, USA).

## Results

### Effect of Sanzi Formula on Body Weight, Size, and Number of Colorectal Adenomas in Mice

**Figure 1.**
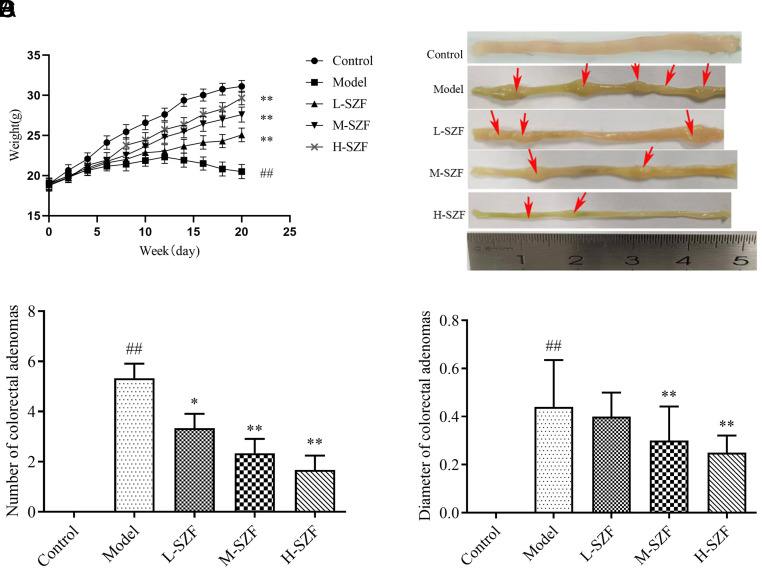
Effect of SZF on body weight and colorectal adenomas in mice. A. Body weight. B. Histomorphology of colorectal adenomas. C. Number of colorectal adenomas. D. Diameter of colorectal adenomas. ^##^*P* < .01, compared with the control group; ^*^P < .05, ^**^P < .01, compared with the model group.

Figure 1A showed that model mice had significantly lower body weights than the control group. After treatment with SZF, the body weight increased significantly. Figures 1B-1D showed that model group mice had many visible adenomas in their colorectum. These adenomas were large in diameter and densely clustered. The L-SZF, M-SZF, and H-SZF groups demonstrated a substantial reduction in the number of colorectal adenomas (*P* < .05, *P* < .01). Furthermore, the diameters of the colorectal adenomas were significantly diminished in both the M-SZF and L-SZF groups (*P* < .01). The L-SZF group of mice exhibited a trend toward reduced diameters of colorectal adenomas, but this difference was not statistically significant.

### Effect of Sanzi Formula on Histomorphologic Changes of Colorectal Adenomas in Mice

**Figure 2.**
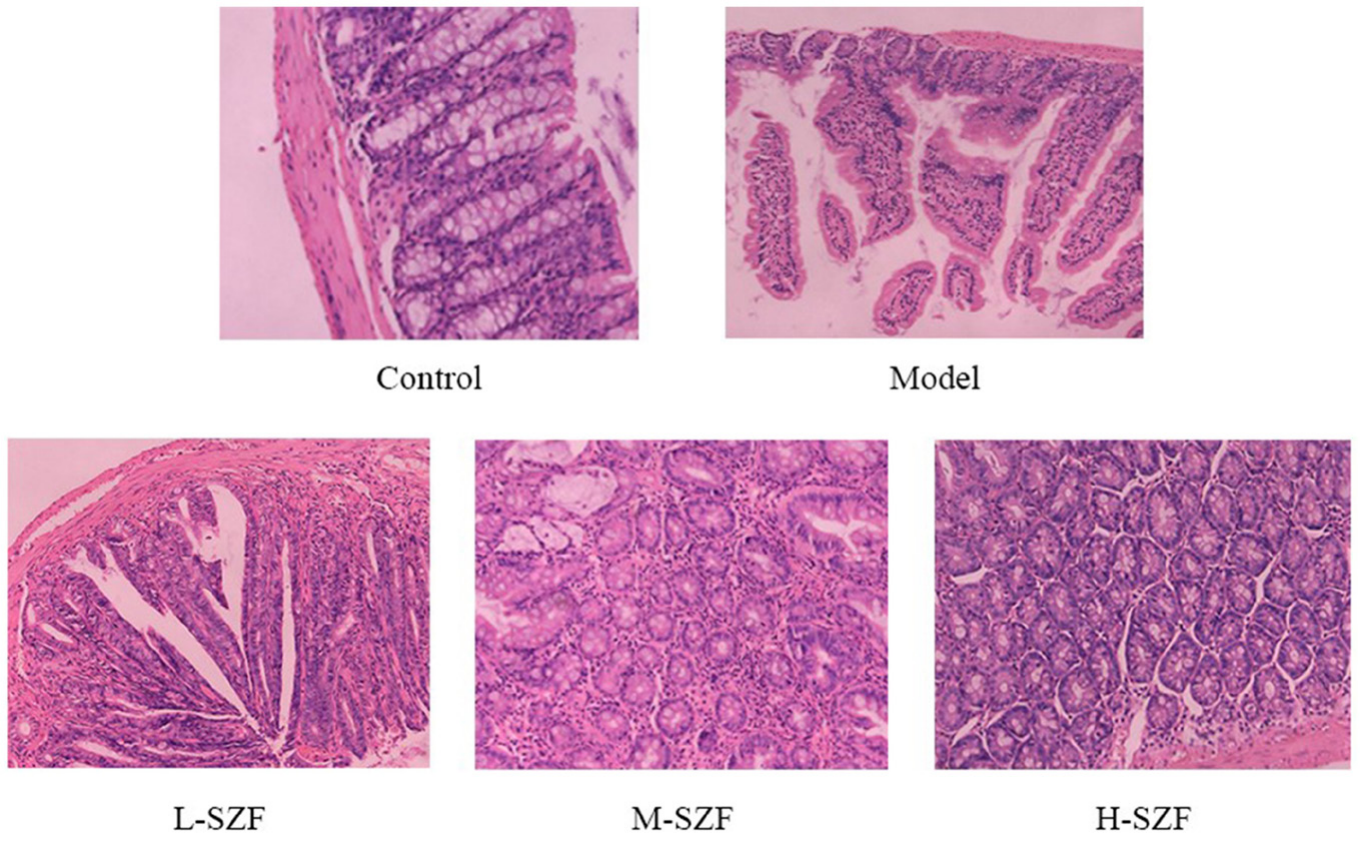
HE staining results of colorectal tissue (×100).

The histopathology of colorectal adenomas in randomly selected samples was observed under the microscope after H&E staining. The colorectal crypts in the control group were structurally intact with normal surface maturation. Pathological results of the model group showed that colorectal tissue presented features of heavy atypical hyperplasia, which was manifested in the cytosolic complex and even occupied the cytoplasm. The glandular ducts exhibited structural abnormalities, characterized by prolongation, twisting, and inconsistent sizes. The phenomenon of shared walls between glandular tubes was widespread, and sieve-like structures were also observed in some areas, with only a few cup-shaped cells present. Pathological findings in the L-SZF group showed that some of the cells were increased in heterogeneity and showed histological heterogeneity, the nuclei of the cells appeared compound, and the apical structures of the cells were still preserved. Additionally, the glandular ducts likewise showed prolonged, twisted, and different sizes, and some ducts exhibited shared walls and back-to-back phenomena. The pathologic tissue from the M-SZF group revealed a decrease in cup cells in the glandular ducts and a slight lengthening of the glandular ducts. The pathologic results of the H-SZF group showed that the cell nuclei were compounded, the apical structures were present, the nuclei were pencil-shaped and tightly arranged, and the glandular ducts were also slightly prolonged (Figure 2). The results showed that different doses of SZF produced different degrees of attenuation of pathological damage to the colorectal tissue of Apc^min/+^ mice. Notably, the H-SZF treatment significantly reduced the severity of atypical hyperplasia in Apc^min/+^ mice and was more effective in preventing colorectal adenoma from becoming malignant.

### Effects of Sanzi Formula on Inflammatory Factors

**Figure 3.**
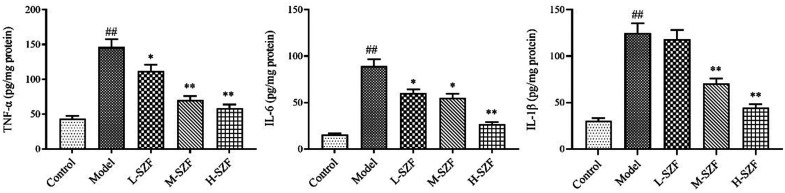
Levels of inflammatory factors TNF-α, IL-6, and IL-1β in colorectal tissue. ^##^*P* < .01, compared with the control group; ^*^P < .05, ^**^P < .01, compared with the model group.

The ELISA assay results demonstrated a significant increase in IL-6, IL-1β, and TNF-α levels in the model group of mice relative to the control group (*P* < .01). The use of SZF significantly reduced these inflammatory markers (*P* < .05, *P* < .01) (Figure 3). The findings indicated that SZF can reduce inflammatory factor levels in mice with colorectal adenoma.

### Effects of Sanzi Formula on Intestinal Barrier Function

**Figure 4.**
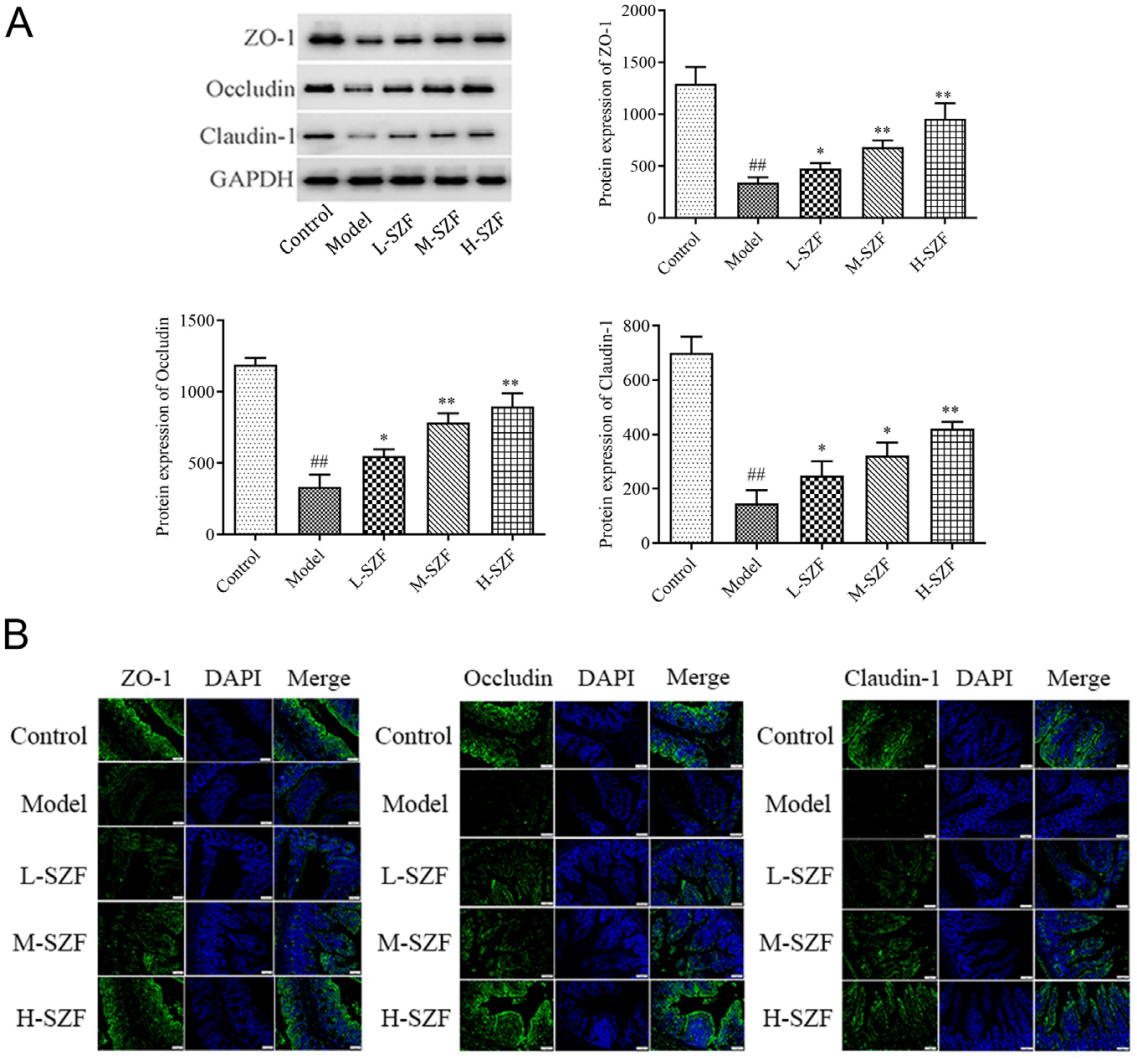
Effect of SZF on the intestinal barrier function. A. Western blot assay was used to detect the protein expressions of ZO-1, Occludin, and Claudin-1 in colorectal tissue. B. DAPI staining of colorectal tissue in immunofluorescence showed ZO-1, Occludin, and Claudin-1 protein expression. ^##^*P* < .01, compared with the control group; ^*^P < .05, ^**^P < .01, compared with the model group.

As illustrated in Figure 4A, the model group exhibited a notable decrease in Occludin, Claudin-1, and ZO-1 proteins compared to the control group (*P* < .01). Conversely, following administration of L-SZF, M-SZF, and H-SZF, the expression of these proteins was significantly elevated relative to the model group (*P* < .05, *P* < .01). The phenomena observed in the immunofluorescence analysis corroborated the findings from Western blotting. As depicted in Figure 4B, the model group displayed diminished levels of Occludin, Claudin-1, and ZO-1 proteins compared to the control group. Nonetheless, treatment with SZF markedly reversed this decline, resulting in a significant boost in the levels of these 3 critical proteins. Collectively, these results suggested that SZF had significantly enhanced the expression of intestinal tight junction (TJ) proteins, thereby improving intestinal barrier function.

### Effects of Sanzi Formula on the Wnt/β-Catenin Signaling Pathway

**Figure 5.**
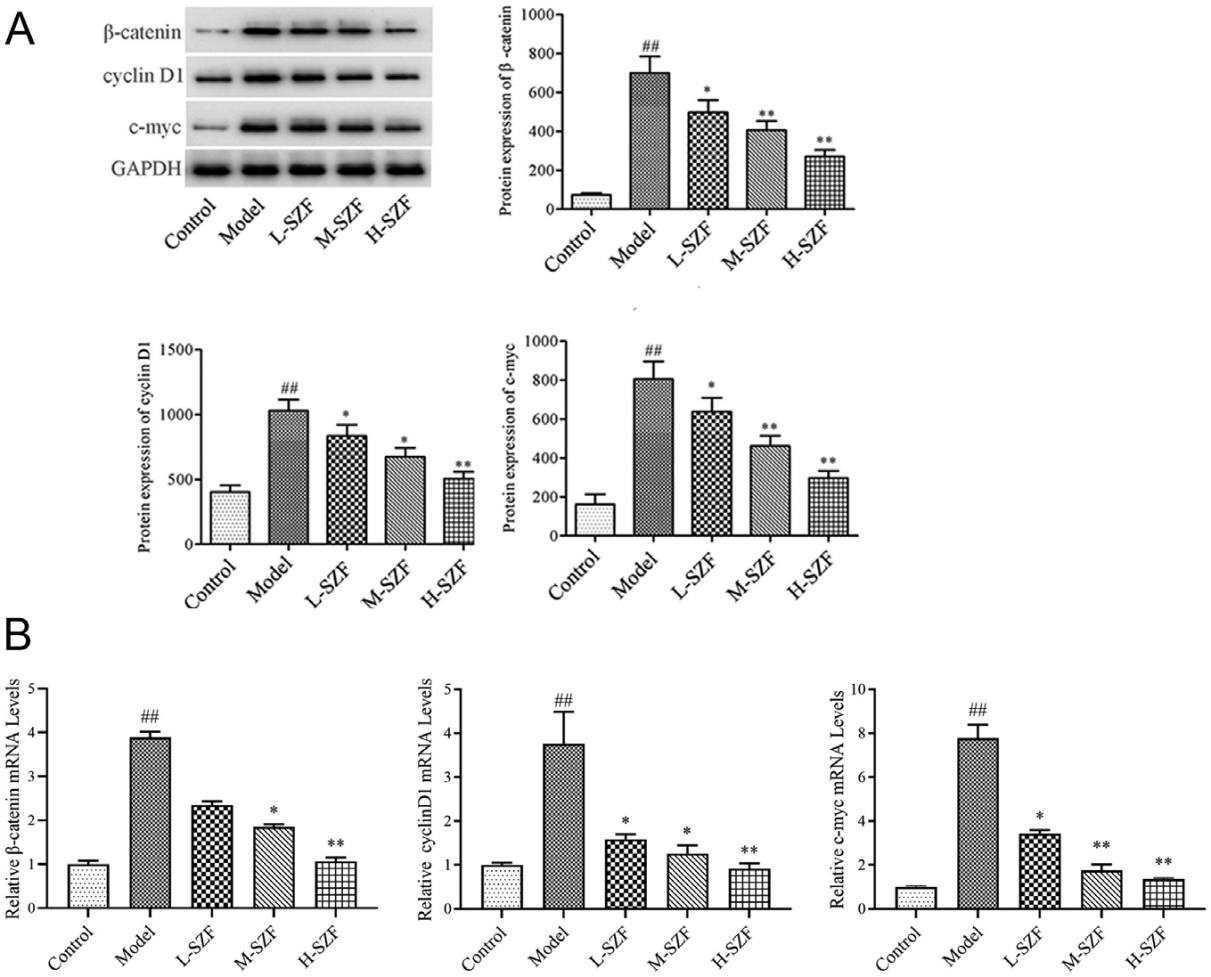
Effect of SZF on the Wnt/β-catenin signaling pathway. A. Western blot detection of β-catenin, cyclin D1, and c-myc protein expressions in colorectal tissue; B. qRT-PCR method was used to detect the expressions of β-catenin, cyclin D1, and c-myc mRNA in colorectal tissue. ^##^*P* < .01, compared with the control group; ^*^P < .05, ^**^P < .01, compared with the model group.

As exhibited by Figure 5A, the model group exhibited significantly greater levels of c-myc, cyclin D1, and β-catenin protein production in contrast to the control group (*P* < .01). The expression of c-myc, cyclin D1, and β-catenin proteins in the L-SZF, M-SZF, and H-SZF groups was notably lower than in the model group (*P* < .05, *P* < .01). As shown in Figure 5B, the model group had larger amounts of c-myc, cyclin D1, and β-catenin mRNA than the control group (*P* < .01). The amounts of c-myc, cyclin D1, and β-catenin mRNA within the L-SZF, M-SZF, and H-SZF groups were considerably smaller as opposed to the model group (*P* < .05, *P* < .01). The outcomes showed the Wnt/β-catenin signaling pathway was blocked by SZF.

## Discussion

Sanzi Formula is composed of *Astragali Radix*,* Mume Fructus*,* Semen Punicae Granati*,* Entadae Semen*,* Phyllanthi Fructus*,* Zanthoxyli Pericarpium*,* Uncariae Ramulus Cum Uncis*,* Curcumae Radix*,* and Glycyrrhizae Radix et Rhizoma*. The main metabolites in *Semen Punicae Granati* include fatty acids, flavonoids, phenolic acids, proteins, volatile oils, etc. and contain pomegranate acid and ellagic acid, which have great potential in the prevention and treatment of cancer.^9^ *Phyllanthi Fructus* is a plant resource with medicinal and food values that has been reviewed and promulgated by the Ministry of Health. Hydrolyzable tannins in the fruits of *Phyllanthi Fructus* are major phenolic compounds with anticancer, antimicrobial, and antioxidant properties.^10^ *Entadae Semen* has the pharmacological effects of promoting qi circulation to relieve pain, purging fire, and detoxification. The saponin element extracted from its seeds has anti-tumor activity.^11^ *Astragali Radix *and* Mume Fructus* can invigorate qi, nourish yin, enhance the function of the liver, and aid the transportation of the spleen and stomach. *Zanthoxyli Pericarpium* warms the middle and relieves pain. *Uncariae Ramulus Cum Uncis* can clear heat, calm the liver, calm the wind, and stop spasms. *Curcumae Radix* can protect the liver and benefit the gallbladder. *Glycyrrhizae Radix et Rhizoma* has pharmacological activities of anti-inflammation, antipyretic, and regulating immune function. This formula can not only invigorate qi and nourish yin but also possesses the efficacy of removing blood stasis and detoxification, which has been clinically proven to have significant efficacy in preventing and treating colorectal adenoma. However, the intervention mechanism of SZF in the progression of colorectal adenoma needs to be explored in depth.

Intestinal inflammation is largely associated with the development of colorectal adenomas.^12^^,^^13^ Prolonged intestinal inflammation leads to a constant inflammatory microenvironment. Inflammatory cells release a variety of cytokines, chemokines, and reactive oxygen species, disrupting the normal immune balance in the gut. Abnormalities in the functioning of immune cells may not be able to effectively recognize and remove abnormal cells, which increases the risk of cellular carcinogenesis.^14^ The sustained activation of the nuclear factor-κB (NF-κB) pathway is associated with inflammation, promoting cell proliferation while inhibiting normal apoptotic mechanisms. This condition may facilitate the malignant transformation of colorectal adenoma. During inflammation, the massive release of reactive oxygen species leads to oxidative stress that damages cellular proteins, lipids, and DNA and promotes malignant cellular transformation. Angiogenic factors secreted by inflammatory cells can provide nutrients and oxygen for tumor cells, accelerating the malignant process of colorectal adenoma. Thus, elevated levels of inflammatory cytokines are considered an important marker of early colorectal cancer development.^15^ Liu et al^16^ found the quantity of inflammatory mediators (IL-6, IL-1β, and TNF-α) in the small intestinal tumors of Apc^min/+^ mice was higher than that in the control group. Wei et al^17^ found a similar increase in blood levels of TNF-α, IL-1β, and IL-6 in a rat model of colorectal cancer. It was also revealed by the investigation that the model group’s colorectal tissue had considerably higher amounts of IL-6, IL-1β, and TNF-α than the control group, aligning with existing literature. This suggested that inflammation is indeed an important driver of colorectal carcinogenesis. Notably, the SZF intervention groups demonstrated a substantial reversal of these elevated cytokine levels, indicating that SZF effectively mitigates inflammatory factors in mice with colorectal adenoma. This finding is an important contribution to this study and further validates the potential of anti-inflammatory drugs in the treatment of colorectal adenoma.

The intestinal barrier function is an important factor in maintaining the stability of the internal intestinal environment. Destruction of the intestinal barrier function triggers microbial community imbalance. The produced metabolites and toxins may affect the gene expression and cell signaling pathways of colorectal cells, thereby promoting the growth of colorectal adenoma. In turn, the process of colorectal adenoma development may further disrupt the intestinal barrier function, creating a vicious cycle that exacerbates intestinal pathology. Various factors, such as stress, high-fat diet, and infection, impair intestinal mucosal barrier function, which is manifested by intestinal epithelial damage, altered mucus layer, and microecological imbalance. This is the early stage during the development of many intestinal diseases, including intestinal tumors.^18^^,^^19^ Tight junctions, an important component of the intestinal mechanical barrier, contain claudins and occludin on the lateral membranes of epithelial cells and ZOs on the inner cytoplasmic membranes.^20^ Abnormal claudin-1 protein expression leads to barrier dysfunction with reduced TJ function and elevated tissue permeability.^21^ This study found that SZF-treated Apc^min/+^ mice had significantly higher colorectal TJ protein levels than the model group. This is similar to the previously reported anticancer properties of berberine, both of which inhibit colorectal carcinogenesis by improving intestinal barrier function.^22^ This indicated that SZF elevated the expression of intestinal TJ proteins, thereby improving intestinal barrier function and effectively inhibiting the growth of colorectal adenomas.

The Wnt/β-catenin signaling pathway activation is complicated by multiple protein interactions. Under normal physiological conditions, β-catenin levels are kept low within cells. Once the Wnt/β-catenin signaling pathway is activated, the associated inhibitory complexes fail, and β-catenin ceases to be degraded and accumulates in the cytoplasm and ultimately in the nucleus. In the nucleus, β-catenin associates with transcription factors such as T-cell factor/lymphocyte enhancer factor (TCF/LEF), thereby activating the expression of downstream target genes, including c-myc and cyclin D1. Abnormal activation of the Wnt/β-catenin signaling pathway increases the risk of colorectal adenoma deteriorating into intestinal cancer.^23^^,^^24^ This study revealed a significant increase in the expressions of c-myc, cyclin D1, and β-catenin proteins in the colorectal tissue of the model group, indicating abnormal activation of the Wnt/β-catenin signaling pathway. This is consistent with previously mentioned results in the literature. Sanzi formula intervention significantly reduced the expression of these 3 proteins and inhibited the Wnt/β-catenin signaling pathway. This study demonstrated that SZF can inhibit the development of colorectal adenomas by modulating the Wnt/β-catenin signaling pathway, offering a novel perspective for research on herbal compounds in anti-tumor applications.

In summary, the findings indicated that SZF may inhibit colorectal adenoma growth by modulating intestinal barrier function and Wnt/β-catenin pathway. However, the present study only investigated the mechanism of action of SZF in inhibiting the development of colorectal adenomas at the animal level. In the future, various models, such as the Caco-2 cell model and organoid model, combined with molecular docking, metabolomics, single-cell sequencing, etc., will be used to further explore the mechanism of SZF in inhibiting the development of colorectal adenoma.

## Figures and Tables

**Table 1.** Gene Primer Sequences for β-catenin, c-myc, and cyclin D1.

Gene	Primers	Nucleotide Sequences 5′-3′	Product Length/bp
β-catenin	Forward	AACAGCAAAGAGCAAATCCAG	106
	Reverse	TGTAGGCAAGAACCATCACAA	
c-myc	Forward	GAAAACGACAAGAGGCGGAC	130
	Reverse	TCACTACCTTGGGGGCCTTT	
cyclin D1	Forward	CAAAATGCCAGAGGCGGATG	109
	Reverse	CATGGAGGGTGGGTTGGAAA	
GAPDH	Forward	CCCAGCTTAGGTTCATCAGG	86
	Reverse	CCAAATCCGTTCACACCGAC	

## Data Availability

The data that support the findings of this study are available upon request from the corresponding author.
